# Identification of a tyrosine switch in copper-haem nitrite reductases

**DOI:** 10.1107/S2052252518008242

**Published:** 2018-06-25

**Authors:** Jianshu Dong, Daisuke Sasaki, Robert R. Eady, Svetlana V. Antonyuk, S. Samar Hasnain

**Affiliations:** a Molecular Biophysics Group, Institute of Integrative Biology, Faculty of Health and Life Sciences, University of Liverpool, Liverpool L69 7ZX, England

**Keywords:** catalysis, redox biology, structural biology, enzyme mechanism, denitrification, nitrogen cycle, copper-haem nitrite reductases, *Ralstonia pickettii*

## Abstract

There are few cases where tyrosine has been shown to be involved in catalysis or the control of catalysis despite its ability to carry out chemistry at much higher potentials (1 V *versus* NHE). Here, it is shown that a tyrosine that blocks the hydrophobic substrate-entry channel in copper-haem nitrite reductases can be activated like a switch by the treatment of crystals of *Ralstonia pickettii* nitrite reductase (*Rp*NiR) with nitric oxide (NO) (−0.8 ± 0.2 V). Treatment with NO results in an opening of the channel originating from the rotation of Tyr323 away from Asp_CAT_97. Remarkably, the structure of a catalytic copper-deficient enzyme also shows Tyr323 in the closed position despite the absence of type 2 copper (T2Cu), clearly demonstrating that the status of Tyr323 is not controlled by T2Cu or its redox chemistry. It is also shown that the activation by NO is not through binding to haem. It is proposed that activation of the Tyr323 switch is controlled by NO through proton abstraction from tyrosine and the formation of HNO. The insight gained here for the use of tyrosine as a switch in catalysis has wider implications for catalysis in biology.

## Abstract 2

Structural characterization of the copper-haem nitrite reductase (haem CuNiR) from *Ralstonia pickettii* (*Rp*NiR) revealed a tyrosine residue blocking the substrate-entry channel and binding site. The treatment of crystals with NO triggers a movement of tyrosine that allows NO- and NO_2_^−^-bound species to be captured to provide the first information on ligand-bound species in this class of nitrite reductases. The use of tyrosine as a switch in activating the redox enzyme *Rp*NiR may have wider significance as this tyrosine is found to be totally conserved in all known haem CuNiRs.

## 1. Introduction   

In biology, redox reactions and catalysis are often performed by redox metals and their cofactors, which typically possess potentials of less than 400 mV. The transition metals iron, copper and manganese are the most utilized redox centres in biology, either on their own or as a component of cofactors such as iron–sulfur clusters, copper–sulfur clusters or haem (Liu *et al.*, 2014 ▸). In some cases redox centres are coupled to orchestrate the delivery of protons and electrons to the catalytic centre for substrate reduction. An example of such an inter-linked mechanism in which the delivery of electrons, substrate and protons are well controlled and regulated through coupled potentials is the well studied copper nitrite reductases (CuNiRs) that catalyse the reaction NO_2_^−^ + e^−^ + 2H^+^ ↔ NO + H_2_O, a step in the microbial ATP-generating denitrification pathway (Maia & Moura, 2014 ▸). The extensive application of nitrogeneous fertilizers has resulted in agriculture being the largest source of atmospheric N_2_O. Denitrifying microorganisms that utilize nitrate as a terminal respiratory electron acceptor produce this potent ozone-depleting and greenhouse gas. Copper-containing nitrite reductase (CuNiR) is a key enzyme in this process since it forms NO, a precursor for N_2_O formation, as the product of the one-electron reduction of nitrite. Electron transfer from a partner cupredoxin or cytochrome redox protein to CuNiR provides the electrons for this reduction.

Structural studies of many CuNiRs have shown that a duplicated cupredoxin-domain monomer unit forms the core of the trimeric enzymes (designated here as two-domain CuNiRs). They contain two types of metal centre: a type 1 Cu (T1Cu) site that accepts electrons from a physiological donor and a catalytic type 2 Cu (T2Cu) centre with His_3_–H_2_O ligation. The two centres are separated by a ∼12.6 Å Cys–His bridge which functions in proton-gated electron transfer (Godden *et al.*, 1991 ▸; Boulanger & Murphy, 2002 ▸; Ellis *et al.*, 2003 ▸; Tocheva *et al.*, 2004 ▸; Antonyuk *et al.*, 2005 ▸; Lawton *et al.*, 2013 ▸). The active-site pocket has an aspartic acid and a histidine residue that are conserved in all CuNiRs and that mutational studies have shown to be essential for effective catalysis (Kataoka *et al.*, 2000 ▸; Boulanger *et al.*, 2000 ▸; Prudêncio *et al.*, 2001 ▸; Ellis *et al.*, 2002 ▸). These residues, designated Asp_CAT_ and His_CAT_, are linked to the catalytic centre *via* a water bridge. The binding of nitrite at the T2Cu centre displaces the H_2_O ligand, induces subtle changes to these residues and significantly increases the reduction potential so as to promote electron transfer from the T1Cu centre, gated by protonation of Asp_CAT_ (Hough, Antonyuk *et al.*, 2008 ▸; Brenner *et al.*, 2009 ▸; Ghosh *et al.*, 2009 ▸; Leferink *et al.*, 2011 ▸). This orchestrated sequence of events minimizes the potential for the formation of a deactivated species with a prematurely reduced T2Cu site from which the solvent-derived water ligand dissociates before nitrite can bind.

Two putative proton-pathway channels at the monomer interface that lead to the T2Cu catalytic site have been identified in two-domain CuNiRs and one has been established to be the substrate-access channel from bulk solvent (Ellis *et al.*, 2003 ▸; Antonyuk *et al.*, 2005 ▸). This channel, which is approximately 6 Å wide, is hydrophobic and is formed by residues from two adjacent monomers. The architecture of this channel has been shown to be important in controlling the coordination geometry of bound nitrite as η^2^-*O*,*O* or end-on η^1^-*O* (Antonyuk *et al.*, 2005 ▸; Fukuda *et al.*, 2014 ▸; Boulanger & Murphy, 2003 ▸) and in determining the rate-limiting step in turnover (Leferink *et al.*, 2014 ▸).

Two new subclasses of CuNiRs have been identified that retain the core structure of the two-domain enzymes but have an extra cupredoxin or cytochrome *c*-containing domain fused at the amino- or carboxy-terminus, respectively. Although they have only recently been recognized, genome analysis shows that both of these classes are widely distributed among Gram-negative α- and γ-proteobacteria isolated from a range of different habitats (Bertini *et al.*, 2006 ▸; Ellis *et al.*, 2007 ▸; Antonyuk *et al.*, 2015 ▸). The first structurally characterized three-domain haem CuNiR, that from *Ralstonia pickettii* (*Rp*NiR; Han *et al.*, 2012 ▸; Antonyuk *et al.*, 2013 ▸), is trimeric, with the haem *c* domain of one monomer in close proximity to the T1Cu site of another monomer that is well placed for effective electron transfer, with a haem–T1Cu separation of 10.6 Å. *Rp*NiR, with its additional tethered cytochrome-containing domain, provides a naturally fused electron-transfer complex, providing an opportunity to compare the roles of specific amino-acid residues in inter-domain electron transfer with transient protein complexes. Comparison of the structure with the binary complex *Ax*NiR–cytochrome *c*_551_ and mutagenesis studies have provided direct evidence for the importance of a hydrogen-bonded water at the haem–cupredoxin domain interface in haem–T1Cu electron transfer, in contrast to the C–C interactions of the binary complex (Antonyuk *et al.*, 2013 ▸; Nojiri *et al.*, 2009 ▸).

The structure of the related three-domain haem-CuNiR from *Pseudoalteromonas haloplanktis* (*Ph*NiR) showed differences in the overall organization of the subunits (Tsuda *et al.*, 2013 ▸). In *Ph*NiR the linker wraps around the neighbouring monomer to reach the distant third monomer to create the cytochrome–catalytic domain interface with an extensive water network as in *Rp*NiR.

Surprisingly, given the retention of the catalytic core architecture of the two-domain NiRs, in both *Rp*NiR and *Ph*NiR, the hydrophobic substrate-access channel is blocked by Tyr323 (Antonyuk *et al.*, 2013 ▸; Tyr313 in *Ph*NiR; Tsuda *et al.*, 2013 ▸), a residue that forms part of the linker between the cytochrome *c* and cupredoxin domains. It had remained a puzzle how the substrate reaches the catalytic site and it has been speculated that the nonfunctional proton channel of the two-domain NiRs might also be used for substrate delivery (Antonyuk *et al.*, 2005 ▸).

## 2. Methods   

### 2.1. Cloning, expression, purification, crystallization and structure determination   

Site-directed mutagenesis was performed using the QuikChange site-directed mutagenesis kit (Agilent). The primers were *Rp*NiR D97N, sense 5′-GCCGCACAACATCACCTGCACGGCGT-3′ and antisense 5′-ACGCCGTGCAGGTTATGTTGTGCGGC-3′. The mutations were confirmed by sequencing before transformation. *Rp*NiR and the *Rp*NiR D97N mutant were expressed in *Escherichia coli* BL21(DE3) cells and purified and assayed as described previously (Han *et al.*, 2012 ▸). For T2D *Rp*NiR the copper-incorporation stage of purification was omitted, resulting in the absence of copper from the T2Cu site. Crystals appeared in 2–3 weeks at 4°C. *Rp*NiR–NO complexes were obtained by treating crystals with NO. An *Rp*NiR D97N–NO crystal was incubated in 100 m*M* sodium nitrite in reservoir solution to obtain the *Rp*NiR D97N–NO_2_^−^ complex. The crystals were cryoprotected in the reservoir solution with 10% glycerol and flash-cooled in liquid nitrogen. Diffraction data were collected from single crystals at 100 K on the I04, I04-1 and I02 PX beamlines at Diamond Light Source. X-ray data were processed with *XDS* (Kabsch, 2010 ▸) for the T2D *Rp*NiR and wild-type (wt) *Rp*NiR structures and *MOSFLM* (Battye *et al.*, 2011 ▸) for all other structures and were merged by *AIMLESS* (Evans & Murshudov, 2013 ▸) in the *CCP*4 program suite (Winn *et al.*, 2011 ▸). The *Rp*NiR D97N–NO structure was solved by molecular replace­ment using PDB entry 3ziy (Antonyuk *et al.*, 2013 ▸) as the search model, refined using *REFMAC*5 (Murshudov *et al.*, 2011 ▸) and rebuilt in *Coot* (Emsley *et al.*, 2010 ▸). The *Rp*NiR D97N–NO and wt *Rp*NiR–NO structures were isomorphous to the *Rp*NiR structure which was used as the starting model for the refinement of both structures. Water molecules and ligands were added manually in *Coot*. H atoms were added at riding positions at the end of refinement. The quality of the model was assessed using *MolProbity* (Chen *et al.*, 2010 ▸). Data-collection and refinement statistics are summarized in Table 1 ▸. Channels, tunnels and pores were visualized using *MOLE* (Sehnal *et al.*, 2013 ▸). Structural figures were prepared using *PyMOL* (v.1.8; Schrödinger).

### 2.2. Activity assay   

The nitrite reductase activities of *Rp*NiR and the *Rp*NiR D97N mutant were measured using an NO electrode with ascorbate-reduced phenazine methosulfate as the electron donors in a glovebox under a nitrogen atmosphere, as described previously (Han *et al.*, 2012 ▸).

## 3. Results   

**Figure 1:**
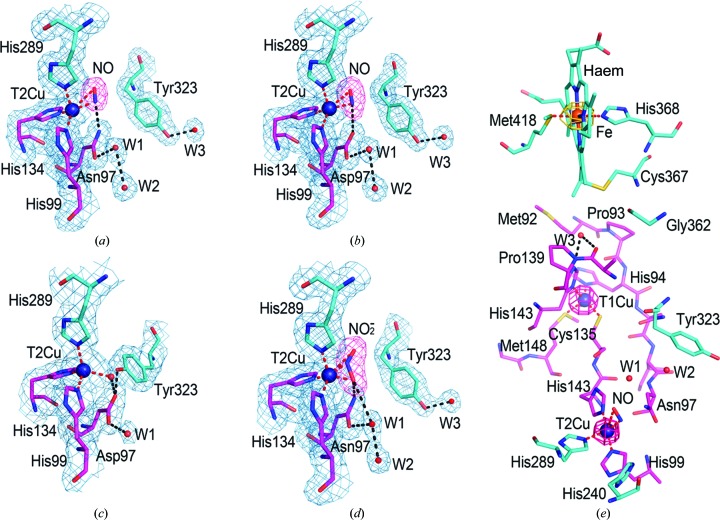
Details of the T2Cu sites of *Rp*NiR and its D97N mutant. (*a*) *Rp*NiR D97N–NO, (*b*) wt *Rp*NiR–NO and (*c*) wt *Rp*NiR without NO treatment in the same space group *I*2_1_3. In both NO-bound structures Tyr323 is rotated away from the T2Cu site compared with the wild-type structure, where it hydrogen-bonds to an aspartic acid residue. W3 is not visible in the wild type here owing to limited resolution, but is well defined in high-resolution structures. (*d*) *Rp*NiR–NO_2_^−^ shows nitrite bound to T2Cu in an outward-facing manner. In (*a*) to (*d*) 2*F*_o_ − *F*_c_ electron-density maps are shown at 1.0σ (blue mesh) and the *F*_o_ − *F*_c_ OMIT maps of NO_2_^−^ and NO are at 7σ (red mesh). His240 has been omitted for clarity. (*e*) An *F*_o_ − *F*_c_ OMIT anomalous map is shown at the 15σ level around copper (1.33 Å X-ray wavelength; red mesh) and at the 20σ level around iron (1.7 Å X-ray wavelength; orange mesh). Hydrogen bonds and copper-coordination bonds are shown as dashed black and red lines, respectively. The two adjacent monomers that form the catalytic centre are coloured magenta and cyan, respectively.

Mutagenesis and structural studies combined with computational analysis of several two-domain CuNiRs have established a role for the invariant Asp_CAT_ residue in the catalytic pocket in proton donation to bound nitrite and in promoting electron transfer from T1Cu to the T2Cu site (Brenner *et al.*, 2009 ▸; Ghosh *et al.*, 2009 ▸; Leferink *et al.*, 2011 ▸). Comparative analysis of the peptide sequences of cytochrome-fused and cupredoxin-fused three-domain NiRs showed that the corresponding aspartic acid residue was conserved (Supplementary Fig. S1), suggesting a similar role for Asp97 of *Rp*NiR in catalysis. We constructed and purified the *Rp*NiR D97N variant enzyme. The normal incorporation of iron and copper and the proper reconstitution of the catalytic T2Cu site were evident from an anomalous diffraction map of the enzyme crystals (Fig. 1 ▸ *e*) and spectroscopic analysis. The electron paramagnetic resonance (EPR) spectrum of the D97N mutant was found to be identical to that of wt *Rp*NiR, showing that both the T1Cu and T2Cu centres were oxidized. As for wt *Rp*NiR, the EPR spectrum remained invariant with nitrite, indicating a lack of binding to the catalytic site or its immediate surroundings. The *Rp*NiR D97N variant enzyme was found to be inactive, as expected owing to impaired proton delivery to the active site.

Despite the fact that the structures of two different haem CuNiRs were published five years ago (Antonyuk *et al.*, 2013 ▸; Tsuda *et al.*, 2013 ▸), no nitrite-bound structures have been reported. Our own efforts to obtain nitrite-bound structures with both wt *Rp*NiR and the D97N mutant failed. In the course of experiments to test whether the nonfunctional proton channel of the two-domain NiRs might also be used for substrate delivery, we made the unexpected observation that the pre-exposure of crystals of wt *Rp*NiR to NO, which has a potential of −0.8 V, allowed structure determination of the NO-bound enzyme. NO treatment results in the activation of Tyr323 such that the hydrogen bond to Asp_CAT_97 is broken, freeing Tyr323 to move away from the substrate-binding pocket and resulting in opening of the substrate-access channel. Similar treatment of the active-site Asp_CAT_ D97N mutant enzyme in which proton donation to the bound substrate is impaired additionally allowed the first structural determination of a nitrite-bound species of a haem CuNiR.

### 3.1. Structures of wt *Rp*NiR–NO and *Rp*NiR D97N–NO   

**Figure 2:**
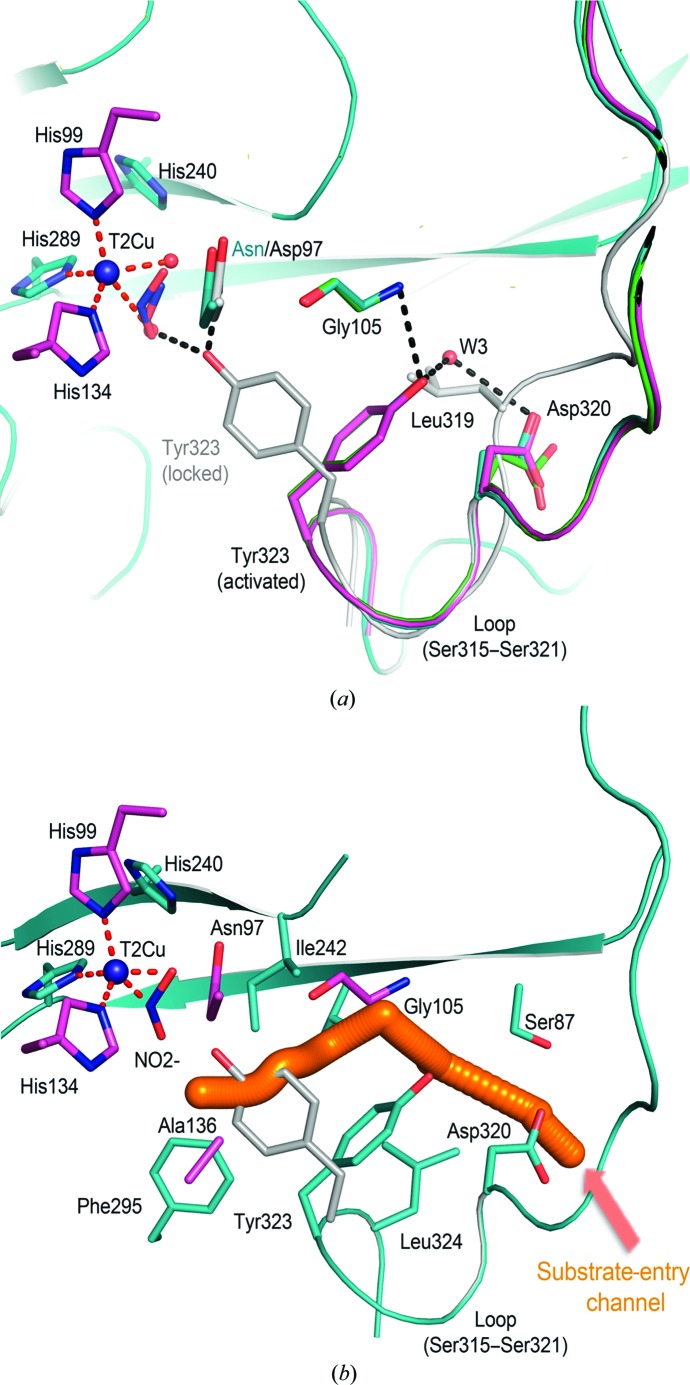
Details of the structural rearrangement accompanying the Tyr323 flip. (*a*) The wt *Rp*NiR–NO (green), *Rp*NiR D97N–NO (cyan) and *Rp*NiR D97N–NO_2_^−^ (magenta) structures show a similar conformation of Tyr323 and loop (Ser315–Ser321) which differs from the free oxidized resting-state wt *Rp*NiR (grey). (*b*) The narrow putative NO-release channel opened by the conformational change, with resting-state Tyr323 shown in grey. T2Cu coordination bonds are coloured red. Water molecules are shown as red spheres.

The structure of as-isolated *Rp*NiR D97N was very similar to that of the wild-type enzyme, including the locked-down position of Tyr323. Exposure of the crystals to NO enabled the structures of NO-bound *Rp*NiR D97N and wt *Rp*NiR to be determined at around 1.8 Å resolution (Figs. 1 ▸ *a* and 1 ▸ *b*). Refinement confirmed that NO was bound to T2Cu in an asymmetric side-on manner with distances of the N_NO_ and O_NO_ atoms to copper of ∼2.0 and ∼2.6 Å in wt *Rp*NiR and ∼2.0 and 2.8 Å in the mutant structure, respectively. The proximity of NO to the side chain of Asp97/Asn97 (Figs. 1 ▸ *a* and 1 ▸ *b*) indicated a probable hydrogen bond (Asp97 O^δ2^/Asn97 N^δ2^ to N_NO_ distance of 3.1 Å). In contrast, NO interacts only weakly if at all with His240, with distances of about 3.5 Å for wt *Rp*NiR and 3.6 Å for *Rp*NiR D97N. The binding of NO results in a 90° flip of the Tyr323 side chain, disrupting a hydrogen bond to Asp_CAT_97 to form a new hydrogen bond to Gly105 N (Figs. 2 ▸ *a* and 2 ▸ *b*). This is accompanied by a large movement of the linker loop, Ser315–Ser321, that connects the haem and cupredoxin domains, resulting in the opening of the blocked channel from bulk solvent to the T2Cu site. As these structures are from a crystal that grew in space group *I*2_1_3, differing from our previously reported structures of wt *Rp*NiR (*H*3 and *P*2_1_3) and mutants (*H*3), the structure of as-isolated wt*Rp*NiR was also determined in space group *I*2_1_3 at 2.3 Å resolution (Fig. 1 ▸ *c*). A comparison of this and all other structures of untreated crystals, including that from another haem CuNiR, *Ph*NiR, shows the invariance of the tyrosine position irrespective of the space group or enzyme. In all cases the tyrosine is in the locked-down position protecting/blocking access to the catalytic copper.

### 3.2. The structure of a substrate-bound haem CuNiR and opening of the substrate-access channel   

We obtained the first substrate-complex structure of *Rp*NiR at 1.89 Å resolution when crystals of *Rp*NiR D97N were pretreated with the product NO before soaking with nitrite. Diffusion of nitrite into NO-primed crystals showed it to bind to T2Cu in a bidentate η^2^-*N*,*O* outward-facing manner, with distances from the N and two O atoms of nitrite to T2Cu of 1.8, 1.9 and 3 Å, respectively (Fig. 1 ▸ *d*). The N-coordination of Cu–NO_2_^−^ that we observe here is the mode favoured by computational chemistry (Solomon *et al.*, 2014 ▸) and has not been seen before in numerous structures of two-domain CuNiRs, in which it is bound through both O atoms.

**Figure 3:**
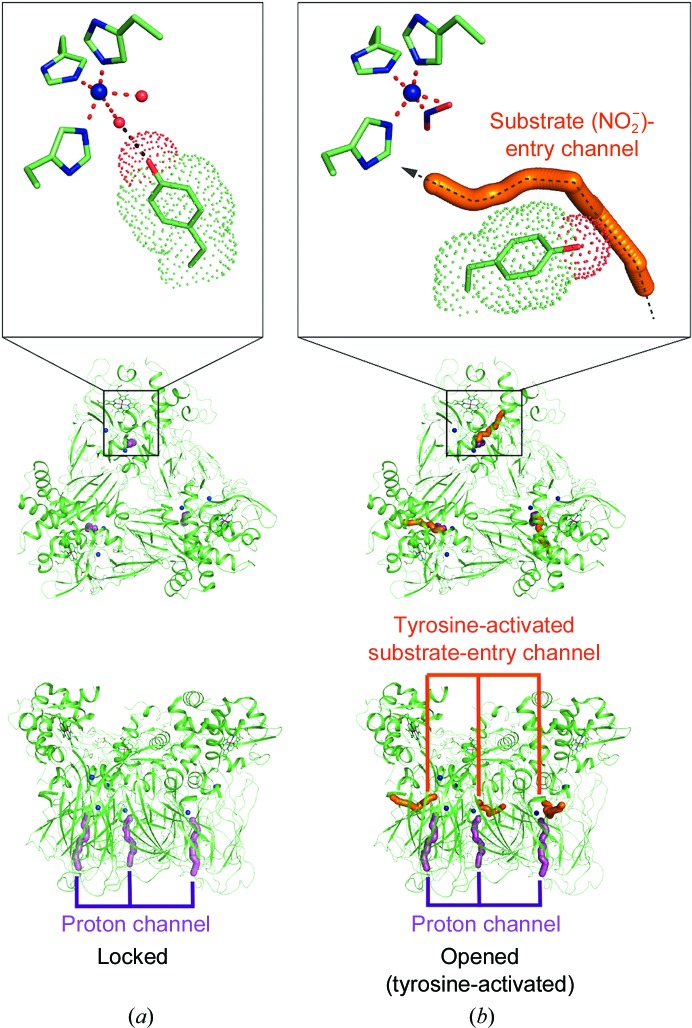
The conformational change leading to the opening of the substrate-access channel. (*a*) Conformation of *Rp*NiR in the resting state showing only the presence of the proton-delivery/product-release channel (magenta). (*b*) Activation of Tyr323 leads to opening of the substrate-access channel (orange) with the proton-delivery channel remaining unaltered. The new channel that is opened as a result of tyrosine activation is the same channel as is used for substrate access in all two-domain CuNiRs.

The substrate-access channel of the two-domain NiRs becomes apparent in the ligand-bound species of wt *Rp*NiR and *Rp*NiR D97N (Fig. 2 ▸). The generation of this channel is a consequence of the new position of Tyr323, which for simplicity is called the ‘activated tyrosine’ position to distinguish it from the proximal ‘locked’ conformation as observed in as-isolated wt *Rp*NiR (Antonyuk *et al.*, 2013 ▸). The substrate channel is narrower compared with two-domain CuNiRs such as *Ax*NiR (Kataoka *et al.*, 2000 ▸; Hough, Eady *et al.*, 2008 ▸) or *Ac*NiR (Antonyuk *et al.*, 2005 ▸). The substrate-binding pocket is well opened in the activated tyrosine position (Fig. 3 ▸). The channel itself is restricted by a hydrophobic filter formed by residues Tyr323, Leu324, Ile242 and Val285 that would play a significant role in controlling the passage of small molecules, including substrate. It is possible that this channel is also used by NO for both the activation of tyrosine and product release.

A movement of the linker loop (Ser315–Ser321) that accompanies the rotation of Tyr323 avoids a potential clash with the side chain of Leu319 (Fig. 2 ▸ *a*). Structural reanalysis of the three-domain haem CuNiR *Ph*NiR (PDB entry 2zoo; Tsuda *et al.*, 2013 ▸) found that the corresponding loop (Thr305–Asn311) was between the locked and activated conformations, and Tyr313 (corresponding to *Rp*NiR Tyr323) is poised to flip open without any steric hindrance (Supplementary Fig. S2). Primary-sequence analysis of different haem CuNiRs from various organisms showed that the corresponding tyrosine is highly conserved (Supplementary Fig. S1). Therefore, the channel identified here (Fig. 3 ▸) is likely to be present in all three-domain haem CuNiRs requiring the activation of tyrosine, and is used for substrate entry in a similar manner to *Rp*NiR and the two-domain CuNiRs.

### 3.3. Structure of reduced wt *Rp*NiR   

Wt *Rp*NiR treated with the strong reductant dithionite showed a colour change from brown to red accompanied by a shift of the Soret band from 408 to 416 nm, indicating reduction of the haem (Han *et al.*, 2012 ▸). This form of reduced crystal only diffracted to a limited resolution of ∼4 Å and the loop (315–325) containing Tyr323 was completely disordered, exposing the T2Cu site. Milder reduction of wt *Rp*NiR crystals by hydroxylamine or ascorbate also resulted in a change of colour, but no structural changes were observed compared with the oxidized structure except for the partial loss of the second water that is linked to Tyr323. In these cases, tyrosine remains in a locked-down position. The addition of NO to solutions of wt *Rp*NiR and the D97N mutant showed no change in the optical spectrum of the haem. Thus, the activation of Tyr323 by NO does not involve reduction or binding of NO to haem. These observations are also consistent with the observation that mild reduction of the enzyme/haem does not activate tyrosine or cause opening of the substrate-access channel.

### 3.4. Structure of T2D *Rp*NiR   

**Figure 4:**
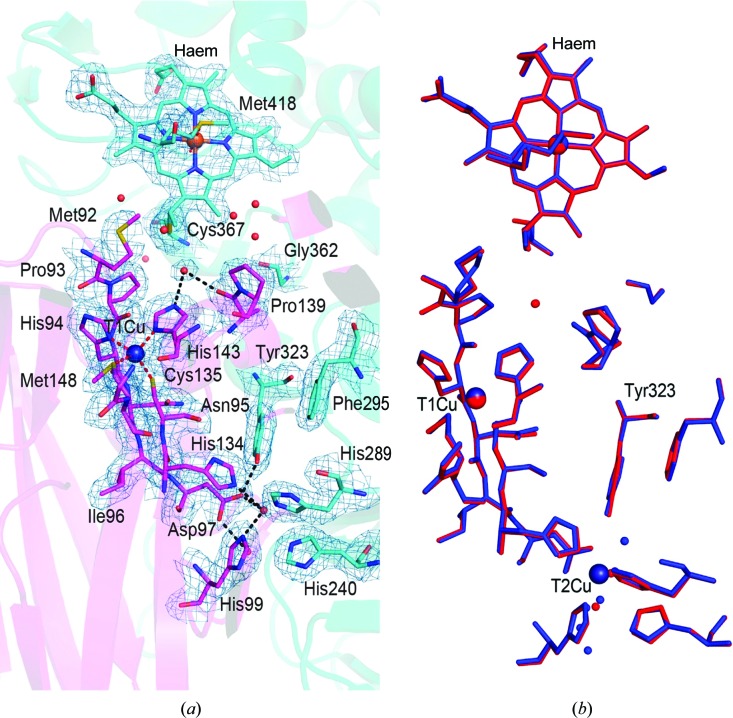
Cytochrome and copper-binding domains in T2D *Rp*NiR and comparison with the fully copper-loaded structure. (*a*) T2D *Rp*NiR has no copper in the T2Cu site. Tyr323 has remained ligated to Asp97 and water and is in the locked position. 2*F*_o_ − *F*_c_ electron-density map is shown at 1.0σ (blue mesh). (*b*) Alignment of the T2D *Rp*NiR structure (red) with the untreated wt *Rp*NiR atomic resolution structure (blue; PDB entry 3ziy) showing close structural similarities, with Tyr323 in an identical position in T2D *Rp*NiR despite the absence of T2Cu.

To determine whether the locked-down conformation of Tyr323 is stabilized by the hydrogen-bonding network of the active-site cavity or by the oxidation state of the T2Cu, we determined the structure of the T2Cu-deficient enzyme at 2.2 Å resolution (Fig. 4 ▸ *a*). In this structure, the side chain of Tyr323 has the same conformation as in the as-isolated wt *Rp*NiR structure. However, the water that usually binds to the phenylate of Tyr323 is absent, but the hydrogen bond (2.6 Å) to the carboxylate of Asp97 is retained. The protein has only one channel connecting the empty T2Cu site to the surface of *Rp*NiR on the dimer interface, similar to that in as-isolated *Rp*NiR. The close similarity of T2D *Rp*NiR to untreated wt *Rp*NiR is clearly evident from a comparison with the atomic resolution structure of the wild-type enzyme that was obtained in the same space group (Fig. 4 ▸ *b*).

## 4. Conclusion   

**Figure 5:**
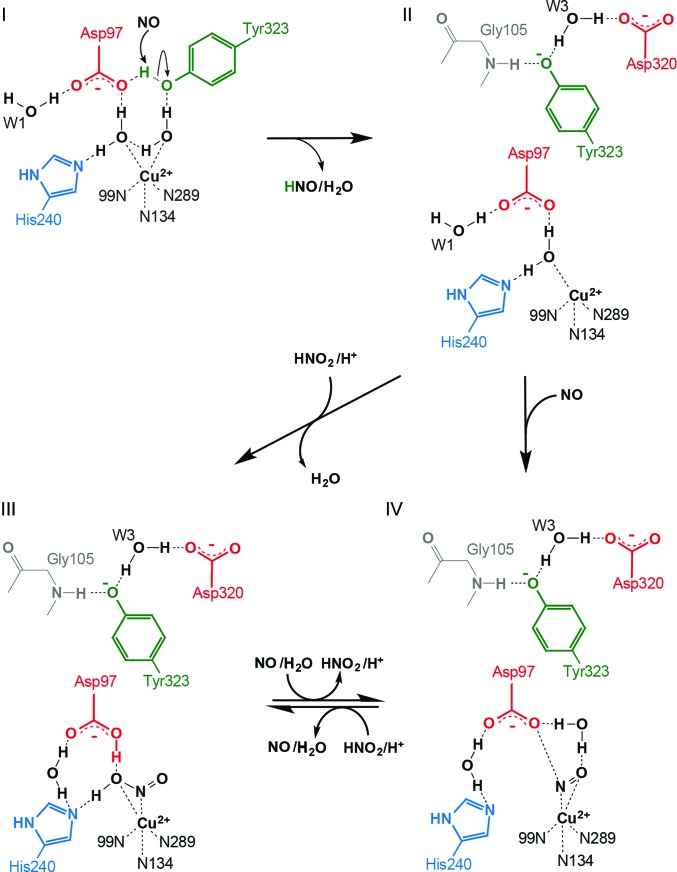
Tyrosine-activation and substrate-binding mechanism of *Rp*NiR. The conformation of Tyr323 is changed by activation with NO by the removal of a water to make hydrogen bonds to a water (W3; hydrogen-bonded to Asp320) and the main-chain N atom of Gly105 (I to II). A substrate (NO_2_^−^) binds to the centre with the removal of a water (II to III). NO binds to the centre of the tyrosine-activated state (II) (II to IV). The formation of the substrate-binding state (III) and the NO-binding state (IV) is reversible. The N atoms of His99, His134 and His289, which are coordinated to T2Cu, are labelled 99N, N134 and N289, respectively.

Our finding that pre-treatment of crystals of *Rp*NiR D97N with NO was required to open the substrate-access channel has enabled structural studies of ligand-bound species in this previously intractable system. We have identified NO-mediated activation of Tyr323, a residue that is invariant in all haem CuNiRs, as a prerequisite to promote this structural change to prime the T2Cu site for ligand binding at the catalytic centre. Both NO- and NO_2_^−^-bound structures of *Rp*NiR D97N revealed rotation of Tyr323 with an accompanying water molecule, and the Ser315–Ser321 loop adopting an open conformation. These movements, which must take place prior to binding of these ligands at T2Cu, would result in a catalytic site that is indistinguishable from the oxidized two-domain NiRs to which nitrite binds with high affinity (Supplementary Fig. S3). The precise mechanism by which nitric oxide, which has a potential of −0.8 V (Bartberger *et al.*, 2002 ▸), activates Tyr323 remains an open question, but what is clear is that it is a design feature of these enzymes that determines substrate entry and the availability of the catalytic site allowing substrate binding. However, our data clearly provide evidence that activation does not involve haem or catalytic copper. We propose that NO disrupts the hydrogen-bonding network around the catalytic site by capturing a proton from Tyr323 in a proton-coupled nucleophilic addition reaction to form HNO and a tyrosine radical, as observed in chemical model systems (Suarez *et al.*, 2015 ▸). This results in the loss of the water bridging Tyr323 with copper, allowing Tyr323 to rotate away from the substrate-binding pocket (Fig. 5 ▸) together with the bridging water, producing a typical catalytic type 2 Cu^2+^ for ligand binding. In all of the ligand-bound structures water remains associated with Tyr323. The observation that the NO_2_^−^-bound structure could be obtained by soaking *Rp*NiR D97N–NO crystals with sodium nitrite suggests a mechanism for substrate binding in which nitrite displaces nitric oxide (step IV to step III in Fig. 5 ▸). A corollary of this would be that once the enzyme is activated, Tyr323 remains in the activated conformation for subsequent turnover just like a switch in the open position.

**Figure 6:**
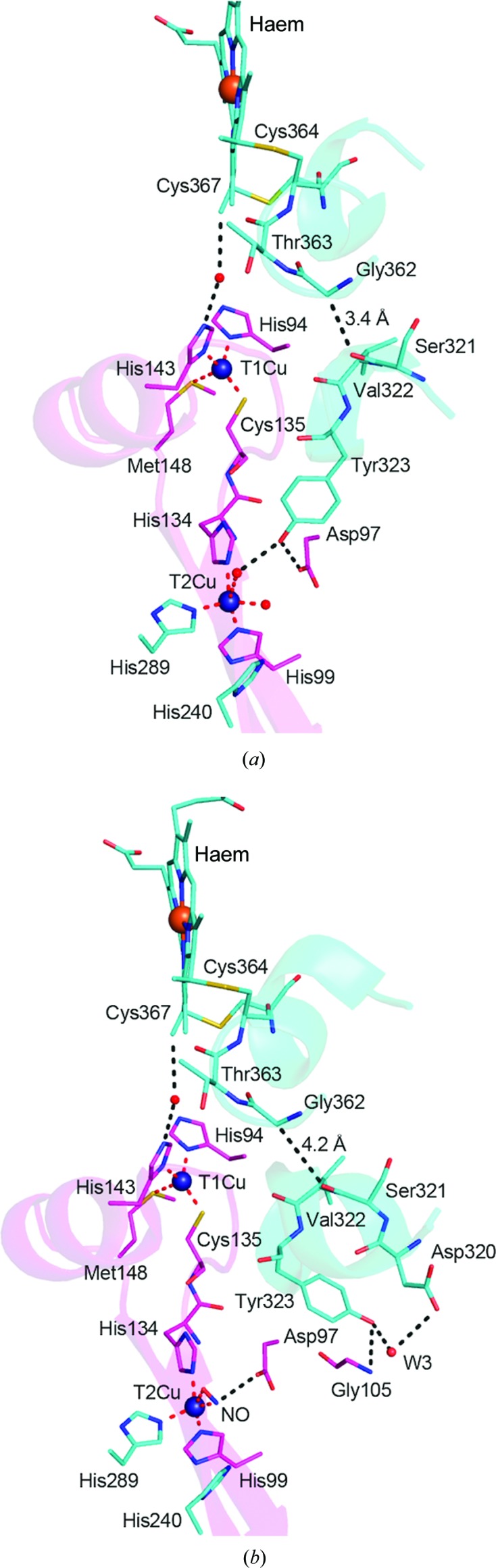
Possible electron-transfer routes from cytochrome to copper centres. (*a*) Wt *Rp*NiR and (*b*) *Rp*NiR–NO. The two adjacent monomers that form the catalytic centre are coloured magenta and cyan. Cu and Fe atoms are shown as blue and orange spheres, respectively, and water molecules as red small spheres. In the wt *Rp*NiR structures two electron-transfer routes appear to be feasible: a through-bond electron-transfer route that leads to Tyr323 *via* Gly362 and water-mediated electron transfer to T1Cu *via* His143. In the ligand-bound structures the water-mediated electron-transfer route to T1Cu remains.

The Tyr323 residue is linked to the haem centre in the cytochrome domain *via* three intervening residues, with the Cys364 residue tethering the haem to the domain. In the as-isolated structures two electron-transfer routes appear to be feasible: a through-bond electron-transfer route that leads to Tyr323 *via* Gly362 and a water-mediated electron transfer to T1Cu *via* His143 (Fig. 6 ▸). In the ligand-bound structures the through-bond contacts to Tyr323 are disrupted, leaving the water-mediated electron-transfer route to T1Cu intact, providing evidence for the function of cytochrome in delivering electrons to the T1Cu site. We propose that T1Cu and T2Cu are coupled in a manner similar to that established for two-domain NiRs, in which a gated mechanism delivers electron transfer to the catalytic copper when substrate nitrite binds (Brenner *et al.*, 2009 ▸; Ghosh *et al.*, 2009 ▸; Solomon *et al.*, 2014 ▸; Hough, Antonyuk *et al.*, 2008 ▸). The role of the through-bond electron-transfer route connecting Tyr323 *via* Gly362 is uncertain but may be involved in the latter stages of reaction.

The functional significance of the fused cytochrome domain in these enzymes has attracted some debate (Antonyuk *et al.*, 2015 ▸). It has been suggested that the additional haem domain of *Ph*NiR might not engage in direct electron transfer to the catalytic core but may have additional or different roles in controlling the specificity towards alternative putative cognate electron-donor proteins (Tsuda *et al.*, 2013 ▸). The data presented here suggest that it does have two roles: firstly the protection of the catalytic T2Cu by Tyr323 that forms part of the linker between the cytochrome and cupredoxin domains and secondly providing electrons to the T1Cu centre for electron-tranfer-gated substrate reduction. The highly conserved nature of tyrosine in 13 haem CuNiRs from various organisms gains further significance from our findings and adds to the widening catalogue of roles that tyrosine plays in biological catalysis (Warren, Ener *et al.*, 2012 ▸; Warren, Winkler *et al.*, 2012 ▸; Glover *et al.*, 2014 ▸; Suga, 2017 ▸). The use of tyrosine activation for the opening of the substrate channel and priming of the substrate-binding pocket may have wider implications for its use for the control and regulation of substrate binding. The involvement of NO in activating tyrosine itself through proton abstraction is intriguing. The use of tyrosine in protecting the catalytic site and its use as a switch in these enzymes is a clear example of the use of tyrosine in controlling/regulating catalysis.

## Supplementary Material

PDB reference: *Rp*NiR D97N–NO_2_^−^, 5obo

PDB reference: *Rp*NiR D97N–NO, 5ocb

PDB reference: wt *Rp*NiR–NO, 5ocf

PDB reference: T2D *Rp*NiR, 6fja

PDB reference: wt *Rp*NiR, 6f1q

## Figures and Tables

**Table 1:** Data-collection and refinement statistics Values in parentheses are for the highest resolution shell.

	*Rp*NiR D97N–NO_2_^−^	*Rp*NiR D97N–NO	Wt *Rp*NiR–NO	T2D *Rp*NiR	Wt *Rp*NiR
Data collection					
DLS beamline	I02	I02	I04-1	I04	I03
Space group	*I*2_1_3	*I*2_1_3	*I*2_1_3	*H*3	*I*2_1_3
Resolution (Å)	80.00–1.89 (1.99–1.89)	90.00–1.78 (1.83–1.78)	90.00–1.80 (1.90–1.80)	46.46–2.20 (2.26–2.20)	127.00–2.30 (2.38–2.30)
*R*_merge_ †	0.12 (0.85)	0.09 (0.65)	0.13 (0.96)	0.08 (0.45)	0.11 (0.73)
〈*I*/σ(*I*)〉	6.7 (1.5)	22 (1.7)	9.7 (1.4)	9.2 (2.1)	13.5 (2.1)
Completeness (%)	99.2 (99.2)	98.4 (99.4)	100 (99.8)	98.9 (90.9)	99.6 (99.2)
Multiplicity	3.4 (3.2)	3.2 (3.2)	6.1 (5.2)	3.3 (2.6)	5.0 (4.9)
Refinement					
Resolution (Å)	66–1.89	60–1.78	78–1.80	46.46–2.20	127–2.30
No. of reflections	74954	88395	92042	86243	41187
*R*_work_/*R*_free_ ‡	0.147/0.175	0.143/0.161	0.149/0.164	0.136/0.17	0.134/0.161
No. of atoms					
Protein	3677	3660	3694	3479	3535
Water	643	790	681	437	442
*B* factors (Å^2^)					
Protein	30.1	24.3	25.8	29.9	46.7
Water	54.2	41.3	45.0	43.2	55.3
R.m.s. deviations					
Bond lengths (Å)	0.014	0.014	0.014	0.011	0.015
Bond angles (°)	1.770	1.492	1.492	1.504	1.670
PDB code	5obo	5ocb	5ocf	6fja	6f1q

† *R*_merge_ = 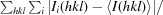 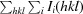, where *I*_*i*_(*hkl*) is the intensity of the measured reflection and 〈*I*(*hkl*)〉 is the mean intensity of all symmetry-related reflections.

‡ *R*_free_ = 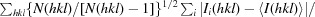 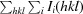 for a test data set of about 5% of the total reflections that were randomly chosen and set aside prior to refinement.

## References

[bb1] Antonyuk, S. V., Eady, R. R. & Hasnain, S. S. (2015). In *Encyclopedia of Inorganic and Bioinorganic Chemistry*, edited by R. A. Scott. New York: Wiley. https://doi.org/10.1002/9781119951438.eibc2316.

[bb2] Antonyuk, S. V., Han, C., Eady, R. R. & Hasnain, S. S. (2013). *Nature*, **496**, 123–126. 10.1038/nature11996 PMC3672994 23535590

[bb3] Antonyuk, S. V., Strange, R. W., Sawers, G., Eady, R. R. & Hasnain, S. S. (2005). *Proc. Natl Acad. Sci. USA*, **102**, 12041–12046. 10.1073/pnas.0504207102 PMC1189323 16093314

[bb4] Bartberger, M. D., Liu, W., Ford, E., Miranda, K. M., Switzer, C., Fukuto, J. M., Farmer, P. J., Wink, D. A. & Houk, K. N. (2002). *Proc. Natl Acad. Sci. USA*, **99**, 10958–10963. 10.1073/pnas.162095599 PMC123192 12177417

[bb5] Battye, T. G. G., Kontogiannis, L., Johnson, O., Powell, H. R. & Leslie, A. G. W. (2011). *Acta Cryst.* D**67**, 271–281. 10.1107/S0907444910048675 PMC3069742 21460445

[bb6] Bertini, I., Cavallaro, G. & Rosato, A. (2006). *Chem. Rev.* **106**, 90–115. 10.1021/cr050241v 16402772

[bb7] Boulanger, M. J., Kukimoto, M., Nishiyama, M., Horinouchi, S. & Murphy, M. E. P. (2000). *J. Biol. Chem.* **275**, 23957–23964. 10.1074/jbc.M001859200 10811642

[bb8] Boulanger, M. J. & Murphy, M. E. P. (2002). *J. Mol. Biol.* **315**, 1111–1127. 10.1006/jmbi.2001.5251 11827480

[bb9] Boulanger, M. J. & Murphy, M. E. P. (2003). *Protein Sci.* **12**, 248–256. 10.1110/ps.0224503 PMC2312428 12538888

[bb10] Brenner, S., Heyes, D. J., Hay, S., Hough, M. A., Eady, R. R., Hasnain, S. S. & Scrutton, N. S. (2009). *J. Biol. Chem.* **284**, 25973–25983. 10.1074/jbc.M109.012245 PMC2757998 19586913

[bb11] Chen, V. B., Arendall, W. B., Headd, J. J., Keedy, D. A., Immormino, R. M., Kapral, G. J., Murray, L. W., Richardson, J. S. & Richardson, D. C. (2010). *Acta Cryst.* D**66**, 12–21. 10.1107/S0907444909042073 PMC2803126 20057044

[bb12] Ellis, M. J., Dodd, F. E., Sawers, G., Eady, R. R. & Hasnain, S. S. (2003). *J. Mol. Biol.* **328**, 429–438. 10.1016/s0022-2836(03)00308-5 12691751

[bb13] Ellis, M. J., Grossmann, J. G., Eady, R. R. & Hasnain, S. S. (2007). *J. Biol. Inorg. Chem.* **12**, 1119–1127. 10.1007/s00775-007-0282-2 17712582

[bb14] Ellis, M. J., Prudêncio, M., Dodd, F. E., Strange, R. W., Sawers, G., Eady, R. R. & Hasnain, S. S. (2002). *J. Mol. Biol.* **316**, 51–64. 10.1006/jmbi.2001.5304 11829502

[bb15] Emsley, P. & Cowtan, K. (2004). *Acta Cryst.* D**60**, 2126–2132. 10.1107/S0907444904019158 15572765

[bb16] Evans, P. R. & Murshudov, G. N. (2013). *Acta Cryst.* D**69**, 1204–1214. 10.1107/S0907444913000061 PMC3689523 23793146

[bb17] Fukuda, Y., Tse, K. M., Lintuluoto, M., Fukunishi, Y., Mizohata, E., Matsumura, H., Takami, H., Nojiri, M. & Inoue, T. (2014). *J. Biochem.* **155**, 123–135. 10.1093/jb/mvt107 24293549

[bb18] Ghosh, S., Dey, A., Sun, Y., Scholes, C. P. & Solomon, E. I. (2009). *J. Am. Chem. Soc.* **131**, 277–288. 10.1021/ja806873e PMC2629382 19053185

[bb19] Glover, S. D., Jorge, C., Liang, L., Valentine, K. G., Hammarström, L. & Tommos, C. (2014). *J. Am. Chem. Soc.* **136**, 14039–14051. 10.1021/ja503348d PMC4195373 25121576

[bb20] Godden, J. W., Turley, S., Teller, D. C., Adman, E. T., Liu, M. Y., Payne, W. J. & LeGall, J. (1991). *Science*, **253**, 438–442. 10.1126/science.1862344 1862344

[bb21] Han, C., Wright, G. S., Fisher, K., Rigby, S. E., Eady, R. R. & Hasnain, S. S. (2012). *Biochem. J.* **444**, 219–226. 10.1042/BJ20111623 22414182

[bb22] Hough, M. A., Antonyuk, S. V., Strange, R. W., Eady, R. R. & Hasnain, S. S. (2008). *J. Mol. Biol.* **378**, 353–361. 10.1016/j.jmb.2008.01.097 18353369

[bb23] Hough, M. A., Eady, R. R. & Hasnain, S. S. (2008). *Biochemistry*, **47**, 13547–13553. 10.1021/bi801369y 19053252

[bb24] Kabsch, W. (2010). *Acta Cryst.* D**66**, 125–132. 10.1107/S0907444909047337 PMC2815665 20124692

[bb25] Kataoka, K., Furusawa, H., Takagi, K., Yamaguchi, K. & Suzuki, S. (2000). *J. Biochem.* **127**, 345–350. 10.1093/oxfordjournals.jbchem.a022613 10731703

[bb26] Lawton, T. J., Bowen, K. E., Sayavedra-Soto, L. A., Arp, D. J. & Rosenzweig, A. C. (2013). *J. Biol. Chem.* **288**, 25575–25583. 10.1074/jbc.M113.484543 PMC3757218 23857587

[bb27] Leferink, N. G., Antonyuk, S. V., Houwman, J. A., Scrutton, N. S., Eady, R. R. & Hasnain, S. S. (2014). *Nat. Commun.* **5**, 4395. 10.1038/ncomms5395 PMC4104443 25022223

[bb28] Leferink, N. G., Han, C., Antonyuk, S. V., Heyes, D. J., Rigby, S. E., Hough, M. A., Eady, R. R., Scrutton, N. S. & Hasnain, S. S. (2011). *Biochemistry*, **50**, 4121–4131. 10.1021/bi200246f 21469743

[bb30] Liu, J., Chakraborty, S., Hosseinzadeh, P., Yu, Y., Tian, S., Petrik, I., Bhagi, A. & Lu, Y. (2014). *Chem. Rev.* **114**, 4366–4469. 10.1021/cr400479b PMC4002152 24758379

[bb31] Maia, L. B. & Moura, J. J. G. (2014). *Chem. Rev.* **114**, 5273–5357. 10.1021/cr400518y 24694090

[bb32] Murshudov, G. N., Skubák, P., Lebedev, A. A., Pannu, N. S., Steiner, R. A., Nicholls, R. A., Winn, M. D., Long, F. & Vagin, A. A. (2011). *Acta Cryst.* D**67**, 355–367. 10.1107/S0907444911001314 PMC3069751 21460454

[bb33] Nojiri, M. Koteishi, H., Nakagami, T., Kobayashi, K., Inoue, T., Yamaguchi, K. & Suzuki, S. (2009). *Nature*, **462**, 117–120. 10.1038/nature08507 19890332

[bb34] Prudêncio, M., Eady, R. R. & Sawers, G. (2001). *Biochem. J.* **353**, 259–266. 10.1042/0264-6021:3530259 PMC1221567 11139389

[bb37] Sehnal, D., Svobodová Vařeková, R., Berka, K., Pravda, L., Navrátilová, V., Banáš, P., Ionescu, C. M., Otyepka, M. & Koča, J. (2013). *J. Cheminform.* **5**, 39. 10.1186/1758-2946-5-39 PMC3765717 23953065

[bb38] Solomon, E. I., Heppner, D. E., Johnston, E. M., Ginsbach, J. W., Cirera, J., Qayyum, M., Kieber-Emmons, M. T., Kjaergaard, C. H., Hadt, R. G. & Tian, L. (2014). *Chem. Rev.* **114**, 3659–3853. 10.1021/cr400327t PMC4040215 24588098

[bb39] Suarez, S. A., Neuman, N. I., Muñoz, M., Álvarez, L., Bikiel, D. E., Brondino, C. D., Ivanović-Burmazović, I., Miljkovic, J. L., Filipovic, M. R., Martí, M. A. & Doctorovich, F. (2015). *J. Am. Chem. Soc.* **137**, 4720–4727. 10.1021/ja512343w 25773518

[bb40] Suga, M. *et al.* (2017). *Nature*, **543**, 131–135. 10.1038/nature21400 28219079

[bb35] Tocheva, E. I., Rosell, F. I., Mauk, A. G. & Murphy, M. E. P. (2004). *Science*, **304**, 867–870. 10.1126/science.1095109 15131305

[bb36] Tsuda, A., Ishikawa, R., Koteishi, H., Tange, K., Fukuda, Y., Kobayashi, K., Inoue, T. & Nojiri, M. (2013). *J. Biochem.* **154**, 51–60. 10.1093/jb/mvt023 23543476

[bb43] Warren, J. J., Ener, M. E., Vlcek, A., Winkler, J. R. & Gray, H. B. (2012). *Coord. Chem. Rev.* **256**, 2478–2487. 10.1016/j.ccr.2012.03.032 PMC3570191 23420049

[bb42] Warren, J. J., Winkler, J. R. & Gray, H. B. (2012). *FEBS Lett.* **586**, 596–602. 10.1016/j.febslet.2011.12.014 PMC3298607 22210190

[bb44] Winn, M. D. *et al.* (2011). *Acta Cryst.* D**67**, 235–242.

